# Application of a Systematic Oral Health Promotion Model for Pregnant Women: A Randomised Controlled Study

**DOI:** 10.3290/j.ohpd.b3555989

**Published:** 2022-11-08

**Authors:** Wenqi Hu, Yijun Wang, Ruyu Chen, Tingting Pan

**Affiliations:** a Professor, Dental Disease Prevention and Treatment Institute of Huangpu District, Shanghai, China. Project administrator, conceptualisation, methodology, formal analysis, investigation, wrote original draft, revised and edited the manuscript.; b Dentist, Dental Disease Prevention and Treatment Institute of Huangpu District, Shanghai, China. Supervised the project, performed investigations.; c Dentist, Dental Disease Prevention and Treatment Institute of Huangpu District, Shanghai, China. Performed investigations.; d Dentist, Dental Disease Prevention and Treatment Institute of Huangpu District, Shanghai, China. Performed investigations, curated data.

**Keywords:** behaviour modification, intervention effect, oral health, pregnancy, women

## Abstract

**Purpose:**

To explore the effects of oral health promotion management on the improvement of oral healthcare knowledge, attitudes, and behaviours in pregnant women.

**Materials and Methods:**

This randomised study included pregnant women in Shanghai (China) who were randomly assigned to receive oral-health promotion management (intervention group) or no interventions (control group). The primary outcome for this study was overall oral health. The secondary outcomes included oral health awareness and attitudes, oral health knowledge, oral healthcare behaviours, medical visits, and risk factors. The Fourth National Oral Health Questionnaire was self-administered in this study, and oral examinations included caries and periodontal status. Data consistency was assessed by the Kappa coefficient.

**Results:**

After intervention, periodontal outcomes in the intervention group had improved statistically significantly, and the proportion of those without periodontal diseases had statistically significantly increased to 14.4% (p < 0.05). In the intervention group, statistically significant improvements were also observed in the number of active caries (p < 0.001), number of filled teeth (p = 0.014), and community periodontal index (CPI) scores (p < 0.001). Overall, after intervention, pregnant women demonstrated comprehension of the importance of children’s deciduous teeth, and their knowledge of the importance of good oral health had greatly improved. Further, oral healthcare habits in the intervention group also showed statistically significant improvement: 56.8% established the habit of cleaning the tongue every week (p < 0.05) and 39.6% established the habit of regular oral examination (p < 0.05).

**Conclusion:**

Oral healthcare education and promotion management for pregnant women can effectively improve their oral health, knowledge, attitudes, and behaviours of oral health care.

Oral health is vital to good overall health and quality of life. Poor oral health can be painful, affect the ability to eat, be esthetically unpleasing, and reduce an individual’s self-esteem. Pregnancy is a crucial period in a woman’s life, with significant physiological changes which allow the body to adapt to the new situation in order to maintain pregnancy,^12^ including cardiac, renal, respiratory, hormonal and metabolic changes, primarily characterised by markedly elevated levels of estrogen and progesterone.^28^ Pregnancy is a crucial period for women’s oral health as well. However, scant attention has been focused on the value of good oral-health awareness and behaviours during pregnancy. Pregnant women often see changes in their diet and hygiene habits that could lead to susceptibility to various oral diseases. Of note, the increased estrogen levels have been associated with both gingivitis and gingival hyperplasia.^26^ During pregnancy, women are more prone to cavities and periodontal disease.^18,21^ Importantly, oral microbiota was shown to be altered during pregnancy, involving changes in hormones, immune function and systemic factors.^29,32^ The literature suggests that periodontitis during pregnancy is closely associated with pre-term birth, adverse pregnancy outcomes and even a diminished quality of life for women during gestation, constituting an important public health concern.^6,11,22,31^ Offspring of women with poor oral health habits are also at risk for developing caries.^8,20,25^

An increasing number of individuals now realise the importance of oral health in improving their quality of life.^3^ Previous epidemiological studies revealed a caries rate among pregnant women in Shanghai as high as 69.8%, which is higher than the 49.4% recorded in Hefei (Anhui Province, China), in a study by Liu Jing et al.^13^ The caries rate in pregnant women in the United States is 62%-87.2%.^5,33^ Many factors are responsible for such a high caries rate, including lack of oral health awareness and personal living habits. Additionally, an effective adult caries prevention system has not been established. Hormonal changes associated with pregnancy often induce pregnancy-related reactions such as vomiting, which reduces the oral pH, leading to an acidic environment, which further corrodes the dental enamel.^4,19,27^

The development of common oral diseases such as caries and periodontal disease is a chronic process caused by bacteria and microorganisms.^15^ Early prevention of these progressive diseases is imperative to reduce their incidence in pregnant women. Prevention is perhaps the most cost-effective way of addressing oral health. Thus, known risk factors must be tackled as early as possible.^2^ Unfortunately, based upon current findings in China and other countries, pregnant women often do not actively seek oral care and there appears to be an insufficient awareness of the importance of oral health care during pregnancy.

Given the general lack of knowledge related to oral health, providing effective and sound advice in the prenatal care setting is necessary. Therefore, this study aimed to explore the effects of a specifically designed systematic oral-health interventional promotion program in pregnant women in a Chinese regional hospital.

## Materials and Methods

### Study Design and Participants

This randomised controlled study included women undergoing pregnancy examinations in Shanghai Red House Obstetrics & Gynecology Hospital in December 2020. Subjects enrolled by a dentist were randomised as follows: on the 23 working days in December 2020, 10 pregnant women were randomly selected every day, for a total of 230 women. Inclusion criteria included: (a) permanent residence in Shanghai; (b) 20–45 years of age; (c) 8–10 weeks of gestation. Women with systemic diseases, e.g. diabetes, cardiovascular diseases or abnormal results in routine blood, liver and kidney function examinations, were excluded to ensure that these factors had no impact on oral health. The study was approved by the Institutional Review Board of Shanghai Huangpu District Dental Center (approval number: HPYF-2019-1) prior to enrolment. All participants provided written informed consent and could withdraw from the study at any time, to ensure voluntary participation. Data were anonymised to preserve subject confidentiality.

### Randomisation

All eligible patients were randomly assigned to the intervention and control groups using a random number table. The intervention group received oral-health promotion management education, oral-health examinations and questionnaire surveys; the control group only received regular oral-health examinations and questionnaire surveys. No oral health care was provided.

### Intervention

Face-to-face health education was conducted 3 times in the intervention group: (1) first trimester (8–10 weeks), provided by a community school for pregnant women; (2) second trimester (20–24 weeks), provided by obstetricians and gynecologists, with health promotion guidance by a stomatological prophylaxis practitioner; (3) last trimester (28–32 weeks), provided by obstetricians and gynecologists, with health promotion guidance by a stomatological prophylaxis practitioner. Oral healthcare knowledge popularisation videos were sent electronically 4 times during the study period (oral health care in the first, second, and last trimesters, as well as neonatal dental-health knowledge). Education was performed using the standard oral health education textbook Oral Health Care in Pregnant Women and Infants, developed by the Chinese Medical Association in 2017 by instructors who conducted this special oral health education and training for women. Instructors carried out extensive sensitisation of the community on the importance of oral health during pregnancy through the distribution of promotional folders, posters and other forms, as well as by providing community workshops for pregnant women. Stomatological practitioners provided one-to-one oral health instructions to pregnant women and implemented health promotion projects.

These special courses consisted of two parts. The first included information regarding tooth care, caries, and periodontal diseases. The second part included a model to demonstrate the correct use of toothbrushes, toothbrushing method, toothpaste and dental floss, and regular professional oral examinations. Other related health education activities included: (1) distribution of self-designed family oral hygiene instruction manuals; (2) 5–10 visits to salons for pregnant women (during which pregnant women learned about oral health care in a relaxed and pleasant atmosphere, while also providing an interactive platform for pregnant women to communicate); (3) online interaction via 5 interactive WeChat groups established by 110 pregnant women, which were managed by two stomatological prophylaxis doctors; (4) watching self-designed videos of proper tooth care.

The control group only received regular oral health examinations and questionnaire surveys, but no oral health care.

The Fourth National Oral Health Questionnaire was utilised in this study, and oral examinations assessed caries and periodontal status. The questionnaire was self-developed and included the physiological characteristics of pregnant women. This questionnaire was self-administered by participants and queried their knowledge of good oral healthcare during pregnancy.

Oral examinations were performed by non-blinded practitioners specialising in oral public health who had received technical training on theoretical and oral examination by the Shanghai Stomatological Disease Center and who underwent a standard consistency check. During each oral examination, participants were re-examined by another examiner at a re-examination rate of 5%. Re-examination results and initial results were subjected to a consistency check. The Kappa value of caries control was >0.9, and the Kappa value of periodontal pocket depth was >0.7. Pregnant women in the present study underwent oral health examinations and completed questionnaires during the first trimester. During the last trimester (34–36 weeks), oral health was evaluated, and the questionnaire was again administered. A Cronbach’s alpha of 0.740 and a KMO value of 0.752 were obtained for the oral health knowledge of the self-administrative questionnaire.

### Data Collection

Baseline demographic data were obtained from each subject, as well as smoking history and educational level. Results from study questionnaires were collected and collated for later analysis.

### Outcomes

The primary outcome of this study was overall oral health: caries rate in permanent teeth, number of active caries, number of missing teeth, number of filled teeth, and Decayed/Missing/Filled Teeth (DMFT) and periodontal diseases (PD) based on the highest community periodontal index (CPI) score. The secondary outcomes included oral-health awareness and attitudes, oral-health knowledge, oral healthcare behaviours, medical visits, and risk factors.

DMFT was the sum of the numbers of decayed, caries-associated missing, and filled teeth for the population undergoing oral examinations. CPI scoring criteria were defined as: healthy = 0, gingival bleeding = 1, calculus = 2, shallow pockets = 3, and deep pockets = 4, according to the WHO.^ 30^

### Sample Size

Sample size was determined based on epidemiological studies revealing a gingival bleeding incidence of 78.7% and assuming an incidence after intervention of 60%, with α = 0.05 (two-tailed) and a statistical power of 0.8. PASS software (NCSS Statistical Software; Kaysville, UT, USA) was utilised, and the required sample size was determined as 103 + 103 cases. Considering possible loss to follow-up, the sample size was enlarged by 10%, i.e. to 113 individuals per group.

### Statistical Analysis

All data were analysed with SPSS 17.0 (SPSS Statistics for Windows; Chicago, IL, USA). Descriptive data, such means ± SD for continuous variables and frequency or percentage for categorical variables, were tabulated. Descriptive and inferential statistics used the Χ^2^ test. p < 0.05 was considered statistically significant.

## Results

The datasets used and/or analysed during the current study are available from the corresponding author on reasonable request.

### Baseline Characteristics

**Fig 1:**
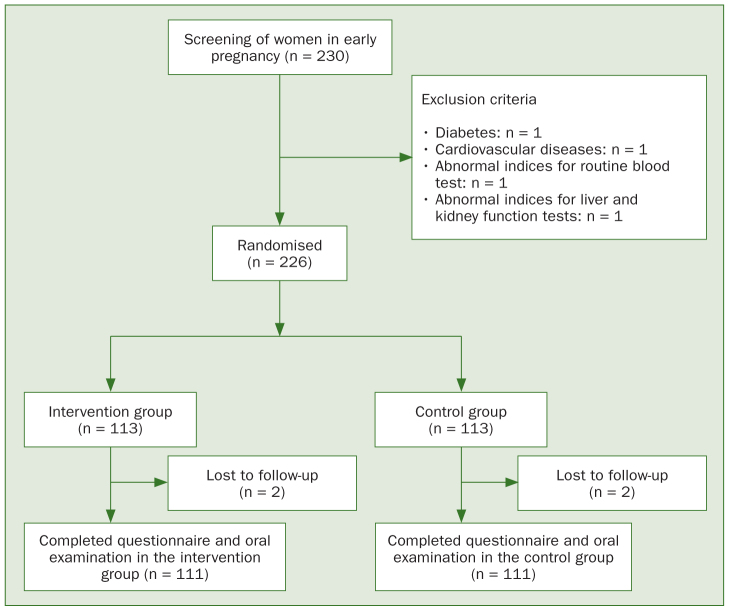
Study flowchart.

A total of 230 pregnant women provided signed informed consent and were included in the study. Several subjects were later excluded due to: diabetes mellitus (n = 1), cardiovascular disease (n = 1), abnormal blood routine index (n = 1), and abnormal liver and kidney functions (n = 1). The remaining 226 subjects were assigned to the intervention and control groups (n = 113). An additional 2 subjects in each group were lost to follow-up. The final analysis included 111 women in each group. The study flowchart is provided in Fig 1. There were no statistically significant differences in baseline characteristics between the two groups in terms of age, smoking history, reproductive history, and educational level (all p > 0.05), as shown in Table 1.

**Table 1:** Baseline characteristics of the control and intervention groups

Characteristic	Control (n = 111)	Intervention (n = 111)	p
Age in years, mean ± SD	30.39 ± 3.948	30.67 ± 4.461	0.622
Smoking history, n (%)	5 (4.5)	1 (0.9)	0.214
Reproductive history, n (%)	25 (22.5)	25 (22.5)	>0.999
**Educational level, n (%)**			0.089
Senior high school and below	15 (13.5)	5 (4.5)	
Junior college	15 (13.5)	12 (10.8)	
Undergraduate	48 (43.2)	59 (53.2)	
Master’s degree and above	33 (29.7)	35 (31.5)	

### Primary Outcomes

No statistically significant differences were observed in caries rate (67.6% and 64.9% in the control and intervention groups, respectively), periodontal disease rate (3.6% and 14.4%, respectively), and missing tooth number (0% for both groups). In the intervention group, improvements were observed in the number of active caries (p < 0.001), the number of filled teeth (p = 0.014), and CPI score (p < 0.001) (Table 2).

**Table 2:** Post-intervention caries and periodontal health overview between the control and intervention groups

Characteristic	Intervention (n = 111)	Control (n = 111)	p
Caries, n (%)			0.670
Yes	72 (64.9)	75 (67.6)	
No	39 (35.1)	36 (32.4)	
Periodontal diseases, n (%)			0.008
Yes	16 (14.4)	4 (3.6)	
No	95 (85.6)	107 (96.4)	
Number of active caries, median (IQR)	0 (0)	1 (2)	<0.001
Number of missing teeth, median (IQR)	0 (0)	0 (0)	0.634
Number of filled teeth, median (IQR)	1 (4)	0 (1)	0.014
DMFT, median (IQR)	1 (5)	2 (4)	0.664
Highest CPI score, median (IQR)	2 (1)	3 (1)	<0.001

### Secondary Outcomes

Through the performed oral health campaigns, the most prominent change was improved knowledge regarding oral health. Of note, statistically significant differences were observed with regard to “knowledge of bacterial effects upon caries” (89.2% and 97.3% in the control and intervention groups, respectively; p = 0.016), “gingival bleeding can be prevented by the brushing of teeth” (67.6% and 88.3% in the control and intervention groups, respectively; p < 0.001), “dental floss use is vital to good oral health” (73.9% and 92.8% in the control and intervention groups, respectively; p < 0.001), and “fluoride can prevent tooth decay” (64.95% and 88.3% in the control and intervention groups, respectively; p < 0.001), as summarised in Table 3.

**Table 3:** Post-intervention rates of oral health knowledge in the control and intervention groups , n (%)

Oral health knowledge item	Intervention (n = 111)	Control (n = 111)	p
Oral diseases may affect general health	107 (96.4)	107 (96.4)	>0.999
Maternal oral problems can affect fetal health	91 (82.0)	84 (75.7)	0.324
Bacteria can cause gingival bleeding	106 (95.5)	99 (89.2)	0.077
Bacteria can cause dental caries	108 (97.3)	99 (89.2)	0.016
Toothbrushing can prevent gingival bleeding	98 (88.3)	75 (67.6)	<0.001
It is necessary to use dental floss	103 (92.8)	82 (73.9)	<0.001
Calcium supplementation during pregnancy is beneficial to fetal tooth development	106 (95.5)	98 (88.3)	0.049
Hard- and soft-bristled toothbrushes are equally effective in tooth cleaning	97 (87.4)	73 (65.8)	<0.001
Fluoride can protect teeth	98 (88.3)	72 (64.9)	<0.001
Dental problems during pregnancy need timely treatment	89 (80.2)	50 (45.0)	<0.001

Through the oral health campaign, the study subjects’ oral health habits showed improvement. The numbers of subjects who cleaned the tongue more than once weekly (43.2% and 56.8% in the control and intervention groups, respectively; p = 0.044) and performed regular oral examinations (24.3% and 39.6% in the control and intervention groups, respectively; p = 0.014, Table 4) were statistically significantly different.

**Table 4:** Post-intervention daily oral healthcare habits in the control and intervention groups, n (%)

Oral hygiene measure	Intervention (n = 111)	Control (n = 111)	p
Toothbrushing >2 times/day	99 (89.2)	98 (88.3)	0.832
Use of dental floss >1 time/day	38 (34.2)	27 (24.3)	0.105
Rinsing the mouth >2 times/day	52 (46.8)	51 (45.9)	0.893
Cleaning tongue coating >1 time/week	63 (56.8)	48 (43.2)	0.044
Correct toothbrushing	52 (46.8)	50 (45.0)	0.788
Regular oral examination	44 (39.6)	27 (24.3)	0.014

The questionnaire results disclosed that while most subjects in each group understood the need for good overall health and stated that it was a personal responsibility to attend regular dental examinations, a statistically significant difference was observed after intervention in terms of “knowledge of the importance of good oral care in children” (74.8% and 89.2% in the control and intervention groups, respectively; p = 0.005) (Table 5).

**Table 5:** Oral health awareness and attitudes of the control and intervention groups, n (%)

Oral health attitude	Intervention (n = 111)	Controls (n = 111)	p
Oral health is very important to your life	111 (100)	110 (99.1)	>0.999
Regular oral examinations are necessary	110 (99.1)	107 (96.4)	0.366
The quality of your teeth is related to paying attention to your own oral health and your own correct oral healthcare behaviour	103 (92.8)	96 (86.5)	0.123
Prevention of dental disease primarily depends on you	110 (99.1)	110 (99.1)	>0.999
It is important to seek help from your dentist for oral problems during pregnancy	110 (99.1)	110 (99.1)	>0.999
Parents have the primary responsibility for children’s oral health	111 (100)	110 (99.1)	>0.999
Children should have regular oral examinations after one year of age	102 (91.9)	96 (86.5)	0.195
Children’s baby teeth are also a concern	99 (89.2)	83 (74.8)	0.005

## Discussion

The increased frequency of food intake during pregnancy, hormonal changes, and multiple other factors, such as fatigue during gestation leading to poor brushing habits, increase bacterial colonisation and the risk of caries.^16,24^ Lack of early intervention has been linked to poor oral-health status.^9,24^ Additionally, despite developments in science and technology surrounding oral healthcare services leading to safe oral diagnosis and treatment during pregnancy, pregnant women often remain reluctant to seek dental care.^14^ Therefore, it is necessary to provide oral-health education for pregnant women as early as possible in order to improve their oral-health awareness and clinical outcomes by reducing the incidence of dental caries, which the current study aimed to address.

Implementing oral-health education campaigns for pregnant women is the most fundamental and cost-effective comprehensive measure for the prevention of dental caries and periodontal diseases. The present study demonstrated that periodontal conditions in the intervention group statistically significantly improved post-education, and the proportion of cases without periodontal diseases increased to 14.4%. Improvements in the number of active caries, number of filled teeth, and periodontal CPI scores also improved after intervention. Previous baseline epidemiological studies have demonstrated that the oral-health awareness and attitudes of pregnant women in Shanghai were the best in all of China, but suggested that more education should be undertaken to bridge the gap for those who did not achieve ideal oral health.^7^ The present study confirmed this notion by identifying few statistically significant changes between the two groups. The only statistically significant difference observed was that subjects in the intervention group expressed knowledge of the importance of children’s deciduous teeth and how crucial good care was for children’s future oral health. Through the implementation of oral-health promotion management, knowledge regarding oral healthcare greatly improved, suggesting that the systematic oral-health promotion management model was effective.

The construction of an oral-health promotion system for pregnant women is crucial for maintaining good oral health in this vulnerable population.^17^ Ideally, these educational campaigns would be led by dental prevention agencies incorporating community family planning professionals, maternal and child health doctors, and obstetricians and gynecologists from local hospitals. It is important that non-dental professionals be deputised to help prioritise oral health education in the workplace by having them schedule time for health education during the work day for pregnant women to ensure educational compliance. Led by dental prophylaxis healthcare workers, the oral-health education and training of non-dental professionals should be strengthened, and special training lectures should be held regularly for these non-dental professionals to establish open communication channels for the implementation of such vital educational endeavors amongst pregnant women.^1,10^

Currently, oral-health education curricula are problematic for students, given they can be boring and utilise a single teaching method that can result in poor compliance. The present study used an engaging self-designed oral health questionnaire suitable for subjects of different educational levels. Additionally, a set of oral healthcare videos was created to enable pregnant women and their families to appreciate the main risk factors for dental caries and periodontal diseases, and to learn how to apply this knowledge daily. An online health education help group was also established to conduct regular educational online interactions stressing the importance of good oral healthcare habits. The contents of these training campaigns were easy to understand as well as targeted, making them suitable for general application. Since pregnant women have more frequent contact with community family planning professionals, maternal and child health doctors in community health service centers, obstetricians and gynecologists played a vital role in the entire project implementation process, and further provided health education for pregnant women in the intervention group at the outpatient clinic. In the present study, non-dental professionals also played an important role. Dental prevention agencies regularly trained these non-dental professionals to enable them to accurately transmit information. The participation of non-dental professionals made the implementation of the entire project more effective and was a vital link in the oral-health promotion management model for pregnant women.

Women are recommended to consult a professional dental facility for a comprehensive oral examination when preparing for pregnancy in order to establish oral-health charts, identify caries in a timely fashion, and receive any requisite treatments, including regular scaling of the whole mouth and removal of any impacted teeth, prior to pregnancy. In the second trimester, women should receive oral healthcare to ensure timely treatment of any dental diseases. After delivery, regular oral care in dental clinics should be instituted, and continued education regarding the importance of good oral healthcare for infants should be undertaken. This would lay a good foundation for the child’s oral health.

The present study had several limitations. Although the sample size calculated for this study meets the statistical requirements, it does not fully represent real-life scenarios. As such, the current findings should be interpreted with caution. In addition, the collaborative efforts of professional and non-dental professionals in this study are still in development and require further improvement for a better interface. Further, it is unclear whether pregnant women would maintain good oral-health habits without supervision. Thus, the proposed oral-health management model should be refined to further enhance intervention effects.

## Conclusion

The present findings suggest that oral health in pregnant women receiving oral-health promotion intervention can be significantly improved by an educational campaign. Additionally, awareness of oral healthcare knowledge and actual oral-health behaviours changed significantly with the implementation of a systematic oral-health promotion management model. This study found improved oral healthcare knowledge and clinical outcomes for pregnant women after educational intervention. An effective oral-health education and behavioural intervention campaign is feasible during pregnancy.
